# A magneto-DNA nanoparticle system for the rapid and sensitive diagnosis of enteric fever

**DOI:** 10.1038/srep32878

**Published:** 2016-09-08

**Authors:** Ki Soo Park, Hyun Jung Chung, Farhana Khanam, Hakho Lee, Rasheduzzaman Rashu, Md. Taufiqur Bhuiyan, Amanda Berger, Jason B. Harris, Stephen B. Calderwood, Edward T. Ryan, Firdausi Qadri, Ralph Weissleder, Richelle C. Charles

**Affiliations:** 1 Center for Systems Biology, Massachusetts General Hospital, Boston, MA, US; 2 Graduate School of Nanoscience and Technology, Korea Advanced Institute of Science and Technology, Daejeon, Korea; 3 International Centre for Diarrhoeal Disease Research, Bangladesh (icddr,b), Dhaka, Bangladesh; 4 Division of Infectious Diseases, Massachusetts General Hospital, Boston, MA, USA; 5 Department of Medicine, Harvard Medical School, Boston, MA, USA; 6 Department of Pediatrics, Harvard Medical School, Boston, MA, USA; 7 Department of Microbiology and Immunobiology, Harvard Medical School, Boston, MA, USA; 8 Department of Immunology and Infectious Diseases, Harvard T. H. Chan School of Public Health, Boston, MA, USA; 9 Department of Systems Biology, Harvard Medical School, Boston, MA, USA

## Abstract

There is currently no widely available optimal assay for diagnosing patients with enteric fever. Here we present a novel assay designed to detect amplified *Salmonella* nucleic acid (mRNA) using magneto-DNA probes and a miniaturized nuclear magnetic resonance device. We designed primers for genes specific to *S*. Typhi, *S*. Paratyphi A, and genes conserved among *Salmonella enterica* spp. and utilized strongly magnetized nanoparticles to enhance the detection signal. Blood samples spiked with *in vitro* grown *S.* Typhi, *S.* Paratyphi A, *S.* Typhimurium, and *E. coli* were used to confirm the specificity of each probe-set, and serial 10-fold dilutions were used to determine the limit of the detection of the assay, 0.01–1.0 CFU/ml. For proof of principle, we applied our assay to 0.5 mL blood samples from 5 patients with culture-confirmed enteric fever from Bangladesh in comparison to 3 healthy controls. We were able to detect amplified target cDNA in all 5 cases of enteric fever; no detectable signal was seen in the healthy controls. Our results suggest that a magneto-DNA nanoparticle system, with an assay time from blood collection of 3.5 hours, may be a promising platform for the rapid and culture-free diagnosis of enteric fever and non-typhoidal *Salmonella* bacteremia.

Enteric fever is caused by *Salmonella enterica* serovar Typhi and Paratyphi (A, B, C), with the majority of cases caused primarily by *S.* Typhi and *S.* Paratyphi A. Enteric fever is endemic throughout the African and Asian continents, with typhoid fever causing an estimated 21.7 million cases per year and over 200,000 deaths annually, and paratyphoid fever causing over 5 million illnesses per year^1^. Enteric fever control has been complicated by the lack of sensitive and specific diagnostic assays that could be used for clinical management and surveillance programs to assess disease burden within a community and measure effectiveness of various intervention strategies (i.e. vaccination campaigns and water, sanitation, and hygiene programs)^2^. Diagnostic assays that detect the bacteria themselves or host responses to infection are therefore being developed.

The TPTest, an assay that detects antibodies secreted by activated lymphocytes, has shown excellent promise^3^,^4^, overcoming the poor sensitivity and specificity associated with plasma and serum-based antibody assays such as the Widal, Tubex, and TyphiDot assays^2^,^5^. Development of assays that directly detect bacteria have themselves been impeded by i) low bacterial burden present in peripheral blood (0.1–10 colony forming units-CFU/mL of blood), ii) lack of laboratory capacity in endemic settings to perform these assays, and iii) frequent prior antibiotic therapy^2^,^5^,^6^. For bacterial detection, culture of bone marrow aspirates is considered to be the gold standard, with sensitivity and specificity of 95 and 100%, respectively, but is limited due to its invasiveness, technical challenge, and requirement for laboratory capacity^2^,^5^.

New point-of-care technologies that can overcome some of these limitations are needed. Here we describe the use of a rapid magneto-DNA nanoparticle assay that can be used to detect *Salmonella* mRNA directly in the blood of infected individuals (Fig. 1). To increase the sensitivity of our assay, we chose genes within operons that have previously been demonstrated to be expressed in the blood of infected individuals with enteric fever^7^,^8^. In addition, we have included three types of signal amplification during the procedure: asymmetric PCR amplification of the target nucleic acid, enrichment of target sequences by bead capture, and magnetic amplification (i.e. a single magnetic nanoparticle can have an effect on billions of surrounding water particles)^9^. Thus, this assay is a promising platform for the rapid and culture-free diagnosis of enteric fever and non-typhoidal *Salmonella* bacteremia.

## Methods

### Ethics Statement

This study was approved by the Research and Ethical Review Committees of the International Centre for Diarrhoeal Disease Research, Dhaka, Bangladesh (icddr,b) and the human studies committees of Massachusetts General Hospital. The study was conducted according to the principles expressed in the Declaration of Helsinki/Belmont Report. Written informed consent was obtained from all individuals or their guardians prior to study participation.

### Study subject selection and sample collection

Individuals presenting to the International Centre for Diarrhoeal Disease Research, Bangladesh (icddr,b) hospital or the Kamalapur field site of the icddr,b with clinical symptoms of enteric fever were eligible for enrollment if they were 1–59 years of age and had a fever (≥38 °C) of 3–7 days duration without an obvious focus of infection or alternate diagnosis. We collected 5–10 ml of venous blood from participants at enrollment (day 0). Five hundred microliters (0.5 mL) of whole blood was placed immediately into TRIzol (Invitrogen Life Technologies, Carlsbad, CA) at a 1 (blood):1 (TRIzol) volume ratio or 1:3 ratio in TRIzol LS. 3–5 ml of blood was used for blood culture using a BacT/Alert automated system. We sub-cultured positive bottles on MacConkey agar, and identified *S*. Typhi and *S.* Paratyphi A isolates in the blood using standard biochemical tests and reaction with *Salmonella*-specific antisera^10^,^11^. After collecting the initial blood, we treated patients with parenteral ceftriaxone and/or oral cefixime for up to 14–21 days at the discretion of the attending physician. For our analysis, we included 3 patients with confirmed *S*. Typhi bacteremia, 2 patients with confirmed *S.* Paratyphi A bacteremia, and 3 North American controls without infection.

### Bacterial strains, plasmids, and media

We included *Escherichia coli* strain B21 and the following sequenced *Salmonella enterica* serotypes obtained from the *Salmonella* Genetic Stock Centre (Calgary, Alberta, Canada) in our analysis: Typhi (strain CT18), Paratyphi A (strain ATCC 9150), and Typhimurium (strain LT2). We grew bacterial strains in Luria-Bertani (LB) media to mid-log growth phase (OD_600_ 0.45–0.6). For the pathogen-spiked human blood sample preparations, 1 ml of blood from a healthy control was added to 1 mL pelleted culture.; mRNA transcripts were preserved with TRIzol LS.

### Primer and Probe Design

The selection of target genes for *S*. Typhi, *S*. Paratyphi A, and *Salmonella enterica* spp. broadly was based on a transcriptome analysis of *Salmonella enterica* serovars Typhi and Paratyphi A in the blood of infected humans in Bangladesh^7^,^8^. We selected genes that were unique to the organisms we were attempting to discern, and genes in operons that have previously been confirmed to be selectively expressed *in vivo* in the blood stream of infected humans^7^,^8^. To ensure minimal cross-reactivity, selected sequences lacked homology with other known pathogens or human sequences. Primers were designed using Primer3. Oligonucleotide sequences that were complementary to the 5′ end and 3′ end of the target sequence were designed and used as capture and detection probes, respectively.

### cDNA synthesis and PCR amplification

**Figure 2:**
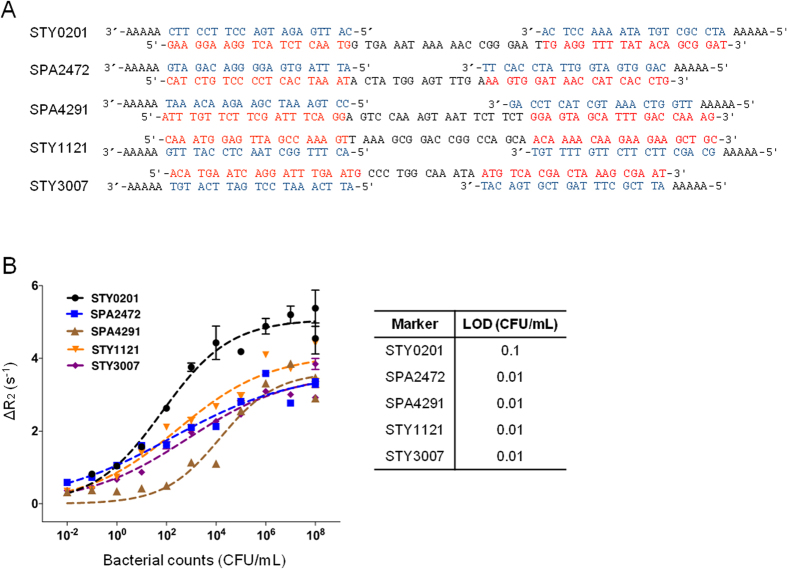
Assay validation using *in vitro* culture. **(A**) Selected primer sets for each target sequence (red). Complimentary oligonucleotide sequences used for the bead capture probe (5′ end of target sequence) and MNP detection (3′end of target sequence) are listed in blue. (**B**) Limit of detection of each primer set in 10 fold serial dilutions of pure *in vitro* grown bacteria.

We extracted total RNA from TRIzol preserved samples per the manufacturer’s instructions (Invitrogen) and RNA was cleaned up and concentrated using Qiagen RNAEasy columns. We then converted up to 1 μg of total RNA into cDNA for each sample using random priming with Promega’s Reverse Transcription System per manufacturer’s protocol, to obtain a representative amplifiable cDNA population. We then amplified target cDNA by asymmetric PCR using primer sets generated for the selected genes listed in Fig. 2A as follows. The cDNA was amplified using Maxima HotStart Polymerase (Fermentas) with reaction volume of 25 μl (10:1 ratio of sense and antisense primers) and 5 μl of cDNA template. The PCR conditions were: 94 °C for 2 min. followed by 50 cycles of denaturation at 94 °C for 5 sec, annealing at 55 °C for 15 sec, extension at 72 °C for 15 sec, and a last extension step at 72 °C for 10 minutes. A positive control (spiked blood) and negative control (unspiked blood) was included in each assay.

### Assessment of probe hybridization

We tested the hybridization of capture and detection probes, before chemical modification, to the target nucleic acids by mixing the probes with the amplified target PCR products derived from *in vitro grown* bacteria (molar ratio of target DNA:capture probe:detection probe = 1:3:3). This mixture was incubated in PBS at 37 °C for 1 hour, and we used polyacrylamide gel electrophoresis to observe retardation of the target nucleic acids.

### Quantification of oligonucleotide probes conjugated onto the beads

The amount of oligonucleotide probes conjugated onto the beads were quantified using the Qubit ssDNA quantification kit (Thermo Fisher Scientific). The conjugated beads were mixed with the Qubit working solution and applied to the Qubit 2.0 Fluorometer (Thermo Fisher Scientific). DNA concentrations were calculated based on a calibration curve using Qubit ssDNA standards.

### Magneto-DNA assay

Microbeads (diameter, 3 μm) were conjugated with capture probes according to the protocol previously reported^9^. Two μL of the PCR reaction solution was mixed with capture probes (4 × 10^6^ beads) and incubated in hybridization buffer (DIG Hyb, Roche Diagnostics) at 37 °C for 20 min. Unbound PCR products were removed through washing the beads with hybridization buffer. Biotinylated detection probes (5 pmol/μL, Integrated DNA Technologies) were added and the mixture was incubated in the hybridization buffer at 37 °C for 20 min. Unbound detection probes were removed through washing with hybridization buffer. For magnetic labeling, streptavidin-coated magnetic nanoparticles (MNPs) (30 nm, 40 μg/mL, Ocean NanoTech) were added and the mixture was incubated in the hybridization buffer at room temperature for 10 min. Unbound MNPs were removed by washing the bead-MNP complexes with hybridization buffer and finally with PBS.

### Target detection with μNMR

The μNMR measurements were done using a previously reported miniaturized μNMR device^12^. In brief, the device has a built-in, automated calibration function to compensate for environmental factors (e.g., temperature, stray magnetic field). The required sample volume is 2 μL, and the sample is loaded into the detection chamber through a disposable plastic tube. We measured transverse relaxation times (*R*_2_) using Carr-Purcell-Meiboom-Gill pulse sequences with the following parameters: echo time, 3 ms; repetition time, 4 s; number of 180° pulses per scan, 900; number of scans, 7. Changes in the transverse relaxation rate (∆*R*_2_) were calculated as *R*_2_ differences between targeted and bead-alone samples. All measurements were in triplicate, and the data are displayed as mean ± standard deviation (s.d.).

### Analytical specificity and sensitivity of the magneto-DNA assay

In order to determine specificity of primers for their respective target serovar, we applied this technique to blood samples spiked with different *Salmonella* serovars and *E. coli.* We determined the sensitivity of the assay using tenfold serial dilutions from 10^8^ to 10^−2^ CFU/mL of pure *in vitro* grown bacteria. We then determined the limit of detection of the assay using human blood samples inoculated with tenfold serial dilutions of bacteria. The limit of detection was determined by the conventional definition: a positive signal exceeded 3 × standard deviation (s.d.) of background signal from samples without bacteria.

### SYBR green-based quantitative real-time PCR (qRT-PCR)

The cDNA derived from *in-vitro* cultured bacteria was mixed with 1x PowerUp SYBR Green Master Mix (Thermo Fisher Scientific) and 0.3 μM specific primers used in the magneto-DNA assay. Thermal cycling was then carried out on 7500 Fast Real-Time PCR system (Thermo Fisher Scientific) with the following conditions: UDG activation (50 °C, 2 min), initiation (95 °C, 2 min); 50 cycles of denaturation (95 °C, 5 s); annealing (55 °C, 15 s); extension (72 °C, 30 s). The 7500 Fast software automatically calculates the Ct value, which represents the first PCR cycle at which the fluorescence signal exceeds the signal of a given uniform threshold.

## Results

### Primer and probe design

We were able to identify *S*. Typhi, *S*. Paratyphi A, and *Salmonella enterica* spp. specific genes that were highly expressed during human infection^7^,^8^ and selected the *S.* Typhi-specific gene, *staG*; the *S.* Paratyphi A-specific genes SPA2472 and *hsdR*, and *Salmonella enterica spp.* conserved genes *sopB* and *sipC*. The primers, sequence-specific targets, and capture and detection probes-specific to the 5′ and 3′ end of each amplified sequence are listed in Fig. 2A.

### Assay Validation

Using total RNA derived from mid-log *in vitro* cultures and polyacrylamide gel electrophoresis, we confirmed the generation of single- and double-stranded (ss and ds) DNA by asymmetric PCR for each primer set (Supplementary Figure S1). We also confirmed the hybridization of the capture and detection probes with the amplified target nucleic acid through observed retardation of the ssDNA of asymmetric PCR products by polyacrylamide gel electrophoresis (Supplementary Figure S1). The number of capture probes per bead was determined to be 150,000~300,000 (Supplementary Table S1). We complexed magneto-DNA hybrids using synthetic target DNA (sequences shown in Fig. 2A), and detected them with the miniaturized μNMR device (Supplementary Figure S1).

### Analytical sensitivity and specificity

**Figure 3:**
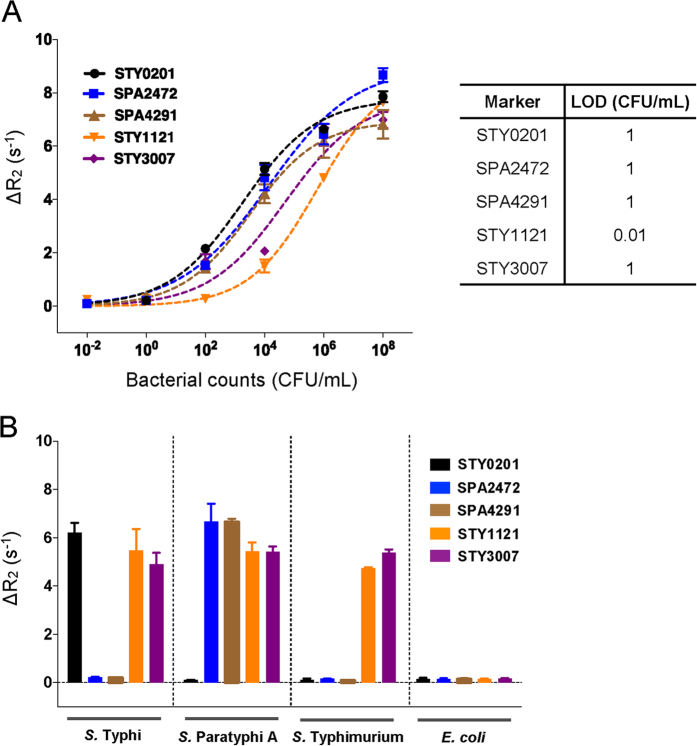
Detection of S*almonella* spiked into human blood. (**A**) Limit of detection of each primer set in spiked blood samples. (**B**) Specificity of each primer set demonstrated in blood samples spiked with different bacteria.

The detection limit of the assay was determined for each primer set across decreasing 10-fold concentrations of *in vitro* grown bacterial CFU/mL and was found to be 0.01–0.1 CFU/mL (Fig. 2B, Supplementary Table S2), which was superior to qRT-PCR (Supplementary Table S3). Once we demonstrated the functionality of the assay with *in vitro* culture, we evaluated the robustness of the assay by detecting decreasing concentrations of *in vitro* grown *Salmonella* in spiked blood samples. The limit of detection of the assay in spiked blood was found to be similar at 0.01–1.0 CFU/mL (Fig. 3A, Supplementary Table S4). We confirmed the specificity of our selected primer sets using blood samples spiked with different serovars of *Salmonella enterica* or *E. coli* as a negative control (Fig. 3B).

### Detection of Salmonella-mRNA from human whole blood

**Figure 4:**
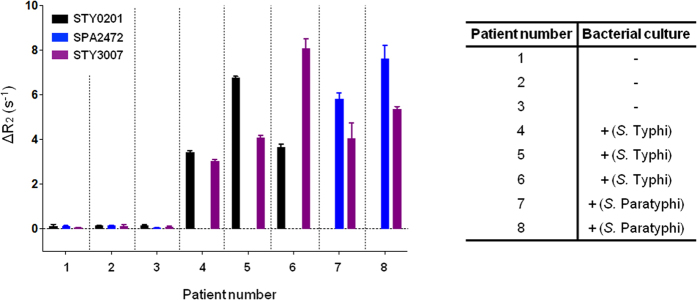
Detection of *Salmonella* in patients with culture-confirmed infection. Detection of *S.* Typhi and *S.* Paratyphi in Bangladeshi patients with blood culture confirmed *S*. Typhi (n = 3) and *S*. Paratyphi A (n = 2) infection and 3 non-endemic healthy controls (North American).

To prove the feasibility of the process, we applied our assay to Bangladeshi patients with culture confirmed *S*. Typhi (n = 3) and *S*. Paratyphi A bacteremia (n = 2) and 3 non-endemic healthy controls (North American). We were able to detect amplified target cDNA using our magneto-DNA assay in all 5 patients with enteric fever, but not in the control samples (Fig. 4). Furthermore, the assay differentiated *S.* Typhi from *S*. Paratyphi A in blood, while all bacteremic samples reacted with the probes conserved in all *S. enterica* serovars.

## Discussion

The availability of a rapid, non-invasive, sensitive and specific technology for detection of *Salmonella* infection during the acute phase of illness is crucial for the clinical management and control of enteric fever. Nucleic acid tests (NATs) are promising given the low bacterial load in the blood of infected individuals, but they have been hampered by low sensitivity, inhibition and contamination issues, and lack of laboratory capacity at sites that are endemic for enteric fever. Here, we have created a rapid diagnostic assay that can detect *S.* Typhi and *S*. Paratyphi A-specific mRNA using a magneto-DNA assay. This assay is more sensitive than standard qRT-PCR in that there are three signal amplification steps of the target sequence in the assay, including PCR amplification, bead capture and enrichment, and magnetic amplification. The method thus does not require pre-enrichment of specimens and in addition can be performed in portable and handheld systems^12^.

Many gene targets for NATs have been used, including the *fliC* gene that is specific for *S*. Typhi, and have shown excellent specificity but variable sensitivity^2^,^13^,^14^,^15^. 16s rRNA has also been used to detect *Salmonella* organisms; however, discerning subspecies *Salmonella enterica* using 16S rRNA NAT has been problematic due to significant sequence homology and requires more advanced PCR techniques^16^,^17^. Similarly, within *Salmonella enterica* serotype Typhi, Paratyphi and non-typhoidal *Salmonella*, housekeeping genes often share significant homology, as well as sharing significant homology across the Enterobacteriaceae, including in *E. coli*. It is for these reasons that we developed this current assay using serotype specific genes and the selected bacterial genes that we have previously shown to be expressed in the blood stream of humans infected with *Salmonella*^7^,^8^. The latter aspect could assist with increasing the likelihood of detection. We therefore designed primer pairs for *S*. Typhi and *S*. Paratyphi A genes in operons that were detected at high levels in the blood of infected humans^7^,^8^ and/or unique to the serotypes that we wanted to detect. We selected a gene that was specific for *S.* Typhi: *staG,* which encodes a fimbrial protein. Two genes were selected that were specific for *S.* Paratyphi A, *hsdR* and *SPA2472. hsdR* encodes a subunit R of a type I restriction modification system and *SPA2472* encodes an uncharacterized protein. We also designed primer pairs for conserved genes specific across the *Salmonella enterica* spp.*, sopB* and *sipC. sopB* is expressed from *Salmonella* Pathogenicity Island (SPI)-5 and encodes a protein involved in creation and maintenance of the *Salmonella* Containing Vacuole (SCV), which is crucial to intra-cellular survival of *Salmonella* in eukaryotic cells^18^. s*ipC* encodes an effector protein of the SPI-1 Type 3 Secretion System involved in invasion of epithelial cells^19^. In our analysis using blood spiked with *in vitro* grown culture, we showed that the limit of detection of our assay is 0.01 to 1 CFU/ml of blood; however, the limit of detection in patient samples may be even better as many of our selected genes had higher expression *in vivo* compared to *in vitro* grown culture^7^,^8^. We also show that our detection system exceeds the level of detection afforded by standard qRT-PCR.

Available NATs are complex and require time-consuming sample preparation. Our analysis is proof-of principle of a method for sample detection of *Salmonella* mRNA in the blood stream of infected patients. The total time for the entire analysis is approximately 3.5 hours from blood collection, significantly less than the 24–48 hours required for microbiologic culture. The current study extends our previous μNMR platform approach^9^ by allowing bacterial serotyping to the subspecies level. Further work is underway to create microfluidic technology designed for RNA sample preparations and cDNA amplification for the rapid detection of *Salmonella* mRNA^20^,^21^.

This assay was able to detect 5 out of 5 cases of confirmed enteric fever and there were no false positives among our 3 North American controls. These results are encouraging, but the clinical cohorts are small. We will need to test our assay in a larger cohort of cases and controls to determine the true sensitivity and specificity of the assay. In addition, the assay was performed in a US-based setting, and we used frozen samples that had varying periods of storage prior to RNA extraction rather than fresh specimens. Validation of our assay will need to be performed in a relevant hospital setting, in order to obtain the true performance characteristics of the test in the field. Nonetheless, our results demonstrate that we can rapidly detect and differentiate *S*. Typhi and *S*. Paratyphi A from non-typhoidal *Salmonella* species in the blood of infected patients.

In conclusion, our currently described μNMR platform approach has improved sensitivity over standard qRT-PCR and identifies *Salmonella* organisms to the subspecies level. Such identification is clinically significant due to differences of the *Salmonella* serotypes in antimicrobial resistance profiles, host characteristics, and control and vaccine program implications. Our results suggest that a magneto-DNA nanoparticle system may be a promising platform for the rapid and culture-free diagnosis of enteric fever and non-typhoidal *Salmonella* bacteremia, as well as for other pathogens present at low levels in the blood.

## Additional Information

**How to cite this article**: Park, K. S. *et al*. A magneto-DNA nanoparticle system for the rapid and sensitive diagnosis of enteric fever. *Sci. Rep.* **6**, 32878; doi: 10.1038/srep32878 (2016).

## Supplementary Material

Supplementary Information

## Figures and Tables

**Figure 1:**
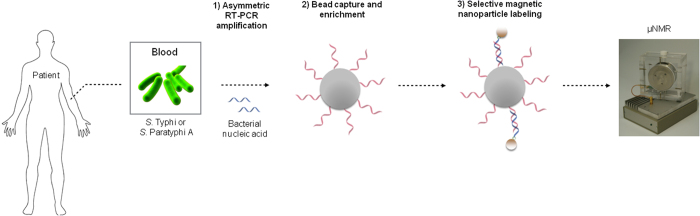
A point-of care nuclear magnetic resonance system for the rapid diagnosis of enteric fever. Schematic representation of detecting pathogen-specific nucleic acid from patients infected with *S.* Typhi or *S*. Paratyphi A using a magneto-DNA nanoparticle system. Total RNA is extracted from a whole blood sample of a patient with suspected enteric fever; the RNA is converted to cDNA and amplified by asymmetric PCR. The amplified target single-stranded DNA is then captured by beads conjugated to capture probes, hybridized with biotinylated detection probes, and then labeled with strepavidin coated MNPs to form a magnetic sandwich complex. Samples are then analyzed using a miniaturized micro-NMR (μNMR) system.
