# Supplementary material for: A magneto-DNA nanoparticle system for the rapid and sensitive diagnosis of enteric fever
Source: Sci Rep. 2016 Sep 8;6:32878. doi: 10.1038/srep32878 (PMC5015101; doi:10.1038/srep32878)
Supplement: Supplementary Information [file srep32878-s1.pdf]

Supplementary information

A magneto-DNA nanoparticle system for the rapid and sensitive diagnosis of enteric fever

Ki Soo Park[1†], Hyun Jung Chung[2†], Farhana Khanam[3], Hakho Lee[1], Rasheduzzaman Rashu[3], Md. Taufiqur Bhuiyan[3], Amanda Berger[4], Jason B. Harris[4,5,6], Stephen B. Calderwood[4,5,7], Edward T. Ryan[4,5,8], Firdausi Qadri[3#], Ralph Weissleder[1,5,9#], Richelle C. Charles[4,5#*]

[1]Center for Systems Biology, Massachusetts General Hospital, Boston, MA, US; [2]Graduate School of Nanoscience and Technology, Korea Advanced Institute of Science and Technology, Daejeon, Korea; [3]International Centre for Diarrhoeal Disease Research, Bangladesh (icddr,b), Dhaka, Bangladesh; [4]Division of Infectious Diseases, Massachusetts General Hospital, Boston, MA, USA; [5]Department of Medicine, Harvard Medical School, Boston, MA, USA; [6]Department of Pediatrics, Harvard Medical School, Boston, MA, USA; [7]Department of Microbiology and Immunobiology, Harvard Medical School, Boston, MA, USA; [8]Department of Immunology and Infectious Diseases, Harvard T. H. Chan School of Public Health, Boston, MA, USA; [9]Department of Systems Biology, Harvard Medical School, Boston, MA, USA; ([†]co-first authors; [#]co-senior authors)

Supplementary Information

Table S1. Quantification of probes conjugated onto beads

**Marker**	**# Oligos/bead (x 10^3)**
STY0201	226 ± 9
SPA2472	271 ± 3
SPA4291	197 ± 4
STY1121	197 ± 8
STY3007	176 ± 13

Table S2. Limit of Detection of assay using *in vitro* grown *Salmonella*

**Marker**	**ΔR_2 (s^{-1}) at LOD**	**ΔR_2 (s^{-1}) at no bacteria**	**3 x SD**
STY0201	0.820	0.679	0.138
SPA2472	0.585	0.483	0.042
SPA4291	0.323	0.211	0.105
STY1121	0.344	0.191	0.045
STY3007	0.369	0.301	0.057

Table S3. Limit of detection of each primer set with serially diluted *in vitro* grown bacteria measured by quantitative real-time PCR. *ND: Not detected, mean Ct ± (s.d.)

		Markers				
		**STY0201**	**SPA2472**	**SPA4291**	**STY1121**	**STY3007**
**Bacterial counts (CFU/mL)**	**No bacteria**	ND	ND	ND	ND	ND
	**10^{-2}**	ND	ND	ND	ND	ND
	**10^{0}**	ND	ND	**ND**	**ND**	**ND**
	**10^{2}**	**ND**	**ND**	41.8 (0.15)	42.5 (0.10)	41.0 (0.67)
	**10^{4}**	40.1 (0.50)	41.0 (0.72)	33.9 (0.42)	34.2 (0.28)	33.8 (0.38)
	**10^{6}**	33.5 (0.65)	34.3 (0.69)	26.4 (0.13)	26.3 (0.28)	25.8 (0.17)
	**10^{8}**	27.1 (0.54)	28.0 (0.44)	20.8 (0.38)	20.6 (0.21)	19.7 (0.41)

Table S4. Limit of detection of assay using *Salmonella* spiked into human blood

**Marker**	**ΔR_2 (s^{-1}) at LOD**	**ΔR_2 (s^{-1}) at no bacteria**	**3 x SD**
STY0201	0.208	0.158	0.048
SPA2472	0.207	0.148	0.045
SPA4291	0.386	0.160	0.060
STY1121	0.252	0.159	0.063
STY3007	0.269	0.162	0.054

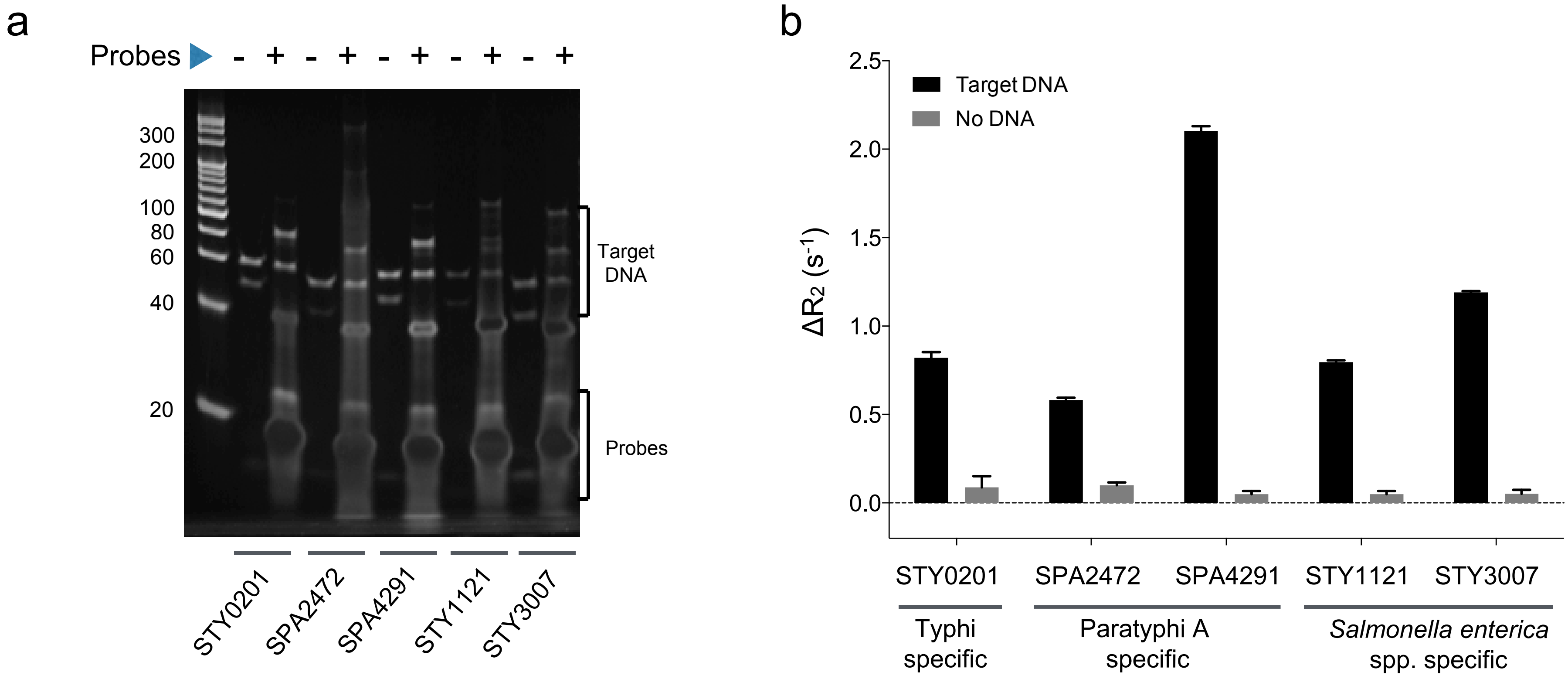

Supplementary Figure S1: Assay Validation. A) The generation of single- and double-stranded DNA by asymmetric PCR was confirmed by polyacrylamide gel electrophoresis. The single-stranded DNA appears below the double-stranded DNA. In the presence of capture and detection probes, there is retardation of the target single-stranded DNA confirming probe-DNA hybridization. B) Detection of magneto-DNA probe hybrids with the miniature NMR device using synthetic oligonucleotides
